# The Pitcher Plant *Sarracenia purpurea* Can Directly Acquire Organic Nitrogen and Short-Circuit the Inorganic Nitrogen Cycle

**DOI:** 10.1371/journal.pone.0006164

**Published:** 2009-07-07

**Authors:** Jim D. Karagatzides, Jessica L. Butler, Aaron M. Ellison

**Affiliations:** Harvard University, Harvard Forest, Petersham, Massachusetts, United States of America; Centre National de la Recherche Scientifique, France

## Abstract

**Background:**

Despite the large stocks of organic nitrogen in soil, nitrogen availability limits plant growth in many terrestrial ecosystems because most plants take up only inorganic nitrogen, not organic nitrogen. Although some vascular plants can assimilate organic nitrogen directly, only recently has organic nitrogen been found to contribute significantly to the nutrient budget of any plant. Carnivorous plants grow in extremely nutrient-poor environments and carnivory has evolved in these plants as an alternative pathway for obtaining nutrients. We tested if the carnivorous pitcher plant *Sarracenia purpurea* could directly take up intact amino acids in the field and compared uptake of organic and inorganic forms of nitrogen across a gradient of nitrogen deposition. We hypothesized that the contribution of organic nitrogen to the nitrogen budget of the pitcher plant would decline with increasing nitrogen deposition.

**Methodology and Principal Findings:**

At sites in Canada (low nitrogen deposition) and the United States (high nitrogen deposition), individual pitchers were fed two amino acids, glycine and phenylalanine, and inorganic nitrogen (as ammonium nitrate), individually and in mixture. Plants took up intact amino acids. Acquisition of each form of nitrogen provided in isolation exceeded uptake of the same form in mixture. At the high deposition site, uptake of organic nitrogen was higher than uptake of inorganic nitrogen. At the low deposition site, uptake of all three forms of nitrogen was similar. Completeness of the associated detritus-based food web that inhabits pitcher-plant leaves and breaks down captured prey had no effect on nitrogen uptake.

**Conclusions and Significance:**

By taking up intact amino acids, *Sarracenia purpurea* can short-circuit the inorganic nitrogen cycle, thus minimizing potential bottlenecks in nitrogen availability that result from the plant's reliance for nitrogen mineralization on a seasonally reconstructed food web operating on infrequent and irregular prey capture.

## Introduction

Nitrogen (N) limits plant growth in most terrestrial ecosystems [1] yet many ecosystems, including arctic tundra [2], coastal salt marshes [3], alpine meadows [4], boreal forests [5], and bogs [6] have large stocks of organic N (ON). More than 90% of soil N is bound in an organic form (humus), 20–40% of this as amino acids [7]. The availability of amino acids may drive ecosystem function in N-limited environments such as arctic tundra because of the very high turnover rates (2–24 h) of amino acids that result from microbial uptake and release [8]. Plants that can use amino acids as an N source may be able to co-exist with, or even outgrow, plants that only use inorganic N (IN), especially in environments where N mineralization rates are low and N limits plant growth [9]–[11]. In Arctic tundra, for example, the most productive species used the most abundant N forms and less productive species used less abundant forms [9].

It has been known for decades that vascular plants can assimilate ON directly when grown in culture [12], with mycorrhizae [13], or in the absence of microbial competition [14], but only in the last decade has ON been shown to be a significant N source for a wide range of plant species in different N-limited systems [15]. Standard theory of N cycling with respect to plant uptake [16] assumes that ON has to be mineralized to IN before it can be assimilated, but direct ON uptake by plants has been proposed to “short-circuit” the N cycle as plants bypass microbial mineralization of ON [17]. This short-circuit is thought to be energetically favourable to plants because ON immediately provides amino acids whereas NH_4_^+^ and NO_3_^−^ (after reduction to NH_4_^+^) must be synthesized into amino acids [18]. Because most ecosystems are N limited and plants can potentially access multiple forms of N in the environment, more information is needed on the generality of direct acquisition of amino acids by plants to fully assess current models of N cycling for a wider range of environments.

Carnivorous plants are generally restricted to extremely N-limited habitats, such as bogs, outwash sand plains, and inselbergs [19]–[21]. In North America, carnivorous pitcher plants (*Sarracenia* spp. and *Darlingtonia californica* Torrey [both in the Sarraceniaceae]) acquire little N from root uptake; up to 80% of their N is obtained from prey captured in their pitcher-shaped leaves [19], [22]–[24]. Most North American pitcher plants secrete chitinases and proteases that directly break down the prey [25], but *S. purpurea* L. secretes digestive enzymes only at very low levels [26] and enzyme secretions have not been observed in *D. californica*. Instead, these two species rely on a food web of aquatic insect larvae, protozoa, and bacteria that inhabits the pitchers [27], [28] to break down the captured prey, mineralize the available ON to IN, and release it for absorption by the pitchers [29], [30]. In northeastern North America where N deposition rates are relatively high, *Sarracenia purpurea* also acquires IN directly from rainfall that collects in its pitchers [31], [32].

This “*Sarracenia* microecosystem” (*S. purpurea* plus its resident food web) has been developed as a model system in which we have examined N cycling of an entire detritus-based food web [22], [30]. In northeastern North America, *S. purpurea* grows in peat bogs and poor fens where plant growth is predominantly N-limited [33]. These bogs have massive stores of ON in peat that is generally assumed to be unavailable for uptake and use by vascular plants, but high nutrient flux and organic production occurs in bogs [6], and many non-carnivorous plants in these habitats have been shown to be able to take up ON (as amino acids) directly through their roots [2], [34], [35]. Carnivorous plants such as *S. purpurea* have weakly developed root systems (root∶shoot ratio ≈0.2) [22], and although carnivorous plants take up some nutrients from their roots, they obtain most of their nutrients from prey captured by modified leaves [36]–[38].

Recent research on the N budget of *S. purpurea* has focused on the relative importance of bacteria and the macroinvertebrates in its food web (larvae of the midge *Metriocnemus knabi* Coq., the mosquito *Wyeomyia smithii* (Coq.), and the sarcophagid fly *Fletcherimyia fletcheri* (Aldrich)) in the nutrient mineralization and excretion process [30]. This work has demonstrated that bacteria are the primary agents of N mineralization, although the mosquito and fly larvae regulate both the abundance and the diversity of the bacteria [39], [40]. Inorganic N derived from atmospheric deposition is directly assimilated by plants [22], [31]. However, neither the ability of pitcher plants to assimilate ON directly, nor the role of the food web in modulating such ON uptake has been investigated experimentally.

Here, we report the results of a 72-hour pulse-chase experiment conducted in the field in which we fed two isotopically enriched amino acids (glycine and phenylalanine) and ammonium nitrate, singly and in combination, to pitcher plants in the field. Our factorial experimental design also assessed whether the macroinvertebrate component of the pitcher-plant's associated food web altered the observed patterns of nitrogen uptake. Finally, we determined if ON uptake by *S. purpurea* and its associated food web differed between sites with different background levels of atmospheric nitrogen deposition.

## Materials and Methods

### Study species

**Figure 1:**
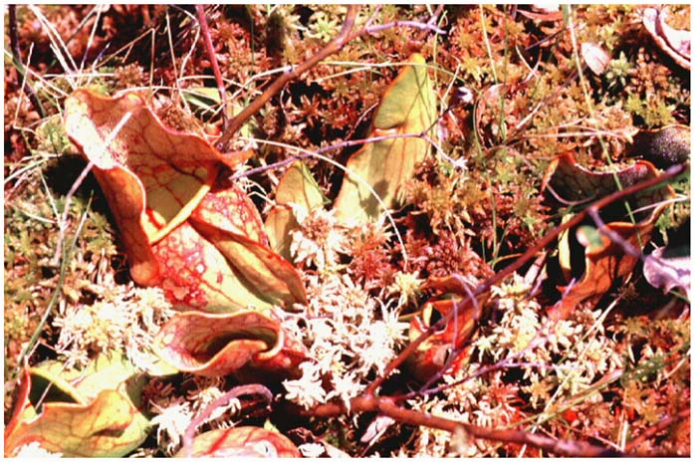
The pitcher plant *Sarracenia purpurea*. This carnivorous plant grows as a rosette of leaves modified into pitchers that act as pitfall traps in which rainfall is collected and prey are captured.

*Sarracenia purpurea* grows in ombrotrophic (rain-fed) bogs [41], poor fens, and seepage swamps throughout Canada east of the Rocky Mountains and in the eastern United States from Maine to Georgia [42]. This long-lived (>50 years), perennial carnivorous plant grows as a rosette of leaves from a small rhizome crown (**Fig. 1**). In the northeastern United States and Canada where we studied *S. purpurea*, it produces 6–10 new leaves each year; the leaves last 1–2 years and then senesce. These leaves are modified into pitfall traps (“pitchers”) that fill with rainwater in which captured arthropod prey drowns. The pitchers also are inhabited by an aquatic, detritus-based food web consisting of bacteria, protozoa, and invertebrates [27], [30], [39]. Prey captured by *S. purpurea* is shredded by aquatic larvae and mineralized by bacteria that inhabit the pitchers; the mineralized nutrients are released for uptake by the plant [29], [30], [43], [44]. *Sarracenia purpurea* is a somewhat inefficient predator – <3% of insect visitors are actually captured [45] – and insects and other prey account for 10–80% of their nutrient budget [23]; it obtains the remainder of its nutrients from stored reserves [22], remobilization and excretion of N and P by rotifers [46], and increasingly, atmospheric deposition [31]. As pitchers generally account for ≈80% of the total plant mass with roots and rhizome crowns accounting for the remaining ≈20% [22], *S. purpurea* derives <5% of its nutrients from the pore water in the peat where it grows [46], [47].

### Field sites

We measured nitrogen acquisition by *S. purpurea* under field conditions at Fort Albany, northern Ontario, Canada (52°15′ N, 81°35′ W) and at Tom Swamp, adjacent to Harvard Pond in Petersham, Massachusetts, U.S.A. (42°30′ N, 72°11′ W). Fort Albany is in the James Bay Lowlands of the Hudson Plains Ecoregion [48], and the dominant vegetation at the study site consists of sedges, mosses, and lichens with or without stunted black spruce (*Picea mariana* (Mill.) Britton, Sterns & Poggenb.) and tamarack (*Larix laricina* (Du Roi) K. Koch). Tom Swamp is a ≈50 ha bog at the north end of Harvard Pond, an artificial pond created in the 1800s by the construction of two dams on Riceville Creek [49]. The bog vegetation is dominated by leatherleaf (*Chamaedaphne calyculata* (L.) Moench.). Fort Albany is near the northern limit of *S. purpurea* in Ontario, but pitcher plants are abundant there as well as at Tom Swamp (densities >5/m^2^). Annual wet inorganic nitrogen deposition for Chapais, Quebec (the nearest data available for Fort Albany and 600 km to the southeast) was ≈2.5 kg/ha in 2002 [48] compared to ≈4.5 kg/ha for central Massachusetts [50].

### Experimental design

We used a 72-hour pulse-chase experiment with isotopically enriched amino acids as our organic nitrogen (ON) source and ammonium nitrate (^15^NH_4_^15^NO_3_) as our inorganic nitrogen (IN) source to determine if pitcher plants can acquire ON directly and to compare ON and IN uptake under different conditions. We focused on uptake of N by pitchers because our previous research showed that pitchers acquired ≈70% of added IN while roots acquired less than 2.5% of added IN [22].

At each site, 125 mature individuals were selected with at least 3 live (no sign of senescence) mature pitchers (firm and open). Five of these plants served as untreated controls and were harvested at the end of the experiment to determine baseline ^15^N and ^13^C natural abundances. The remaining 120 plants were randomly assigned to one of six treatment groups: uniformly-labeled (U-) glycine (**U-Gly**: 98 atom% U-^13^C-^15^N-glycine), uniformly-labeled phenylalanine (**U-Phe**: 98 atom% U-^13^C-^15^N-phenylalanine), **I^15^N** (98 atom% ^15^NH_4_^15^NO_3_), **U-Gly** plus unlabeled phenylalanine and unlabeled NH_4_NO_3_ (hereafter **U-Gly+**), **U-Phe** plus unlabeled glycine and unlabeled NH_4_NO_3_ (hereafter **U-Phe+**), or **I^15^N** plus unlabeled glycine and phenylalanine (hereafter **I^15^N+**). Plants within treatment groups were assigned randomly to one of two harvests (3- or 72-hr) and one of two food web treatments (complete food webs or partial food webs, which lacked the macroinvertebrate larvae of the detritivorous midge *Metriocnemus knabi* and the keystone predator, the mosquito *Wyeomyia smithii*). Larvae of the sarcophagid fly *Fletcherimyia fletcheri*, which are found more commonly in pitchers in Massachusetts than in Canada, were excluded from all experimental pitchers. There were *N* = 5 pitchers in each treatment at each site.

Pitchers were significantly larger at Tom Swamp than at Fort Albany (**Table 1**). Control (unfed) plants at both sites had similar concentrations of N and natural abundance levels of δ^15^N in leaf tissues (**Table 1**). These control plants also had similar C concentrations and similar C∶N ratios, but background natural abundance of δ^13^C was slightly lower at Tom Swamp than at Fort Albany (**Table 1**).

**Table 1:** Comparison of traits for pitchers of untreated plants (*N* = 5) harvested at Fort Albany, James Bay, Ontario, Canada (FA) and Tom Swamp, central Massachusetts, USA (TS) with significance level of unpaired t-test comparing sites.

	Site	Mean	SD	*P*
**Dry Mass (mg)**	FA	232	57	0.00003
	TS	754	142	
**Length (cm)**	FA	8.4	1.9	0.00008
	TS	18.7	1.9	
**δ^15^N (‰)**	FA	1.8	0.8	0.373
	TS	1.1	1.3	
**δ^13^C (‰)**	FA	−26.8	0.7	0.0013
	TS	−29.4	1.0	
**Nitrogen (%)**	FA	1.08	0.13	0.609
	TS	1.02	0.28	
**Carbon (%)**	FA	46.4	3.3	0.408
	TS	47.7	0.3	
**C∶N**	FA	43	4	0.282
	TS	49	10	

Any liquid in the pitchers, along with the food web, was removed from all experimental pitchers the day before the pulse-chase experiment began; the liquid removed (pitcher “liquor”) was kept for the food web manipulations. Following food web removal in the field, pitchers were rinsed with distilled water to remove as much detritus and as many microbes as possible and the pitcher opening was blocked with a fine nylon mesh to limit subsequent entry of animals and prey. In the laboratory, all living midge and mosquito larvae were removed from liquid collected from each pitcher and kept alive overnight in a solution of pitcher liquor.

The next day, the largest pitcher on each plant was fed with one of the ^15^N treatments. We fed each manipulated pitcher with a 0.8 mM ^15^N solution (2 ml for Fort Albany and 9 ml for the larger pitchers at Tom Swamp) and an equal amount of pitcher liquor, resulting in pitchers filled to approximately three-quarters of their volume. Thus, all experimental pitchers contained an enriched (^15^N) nutrient solution along with the microbial component of the food web (supplied in the pitcher liquor). Pitchers at Fort Albany were fed 0.022 mg N, whereas the larger pitchers at Tom Swamp were fed 0.101 mg N. The amount of N added to pitchers represented <1.0% of N content of pitcher tissue at Fort Albany and <1.9% of N content of pitcher tissues at Tom Swamp.

When we added only single forms of N (i.e., the **U-Gly**, **U-Phe**, and **I^15^N** treatments), all N added to the pitchers was enriched in ^15^N. When we added three forms of N (the **U-Gly+**, **U-Phe+**, and **I^15^N+** treatments), only one-third of the N added to each pitcher was enriched in ^15^N; the remaining two-thirds was comprised of equal amounts of the other two forms as unlabeled N. We are confident that we minimized potential effects of excess N availability on pitcher N uptake, which could have been particularly important when only one form of N was added in a single feeding event. The total amount of N supplied and the actual concentration of N were both substantially lower than that used in other studies of *Sarracenia*: 1.2–3.6 mg N/plant as a mixture of amino acids to pitchers of *S. flava* [51]; 1–10 mM alanine fed to *Nepenthes* pitchers [52]; 20 ml/pitcher of 6.8–8.6 mM NH_4_-N (1.9–2.4 mg N/pitcher) [22], [29] or 6–8.7 mg N/pitcher as NH_4_Cl to *S. purpurea* [23], [31], which is comparable to the mass of prey-N captured by *S. purpurea* in a growing season [53].

Finally, for the complete food web treatments, we put invertebrate larvae into the pitchers immediately after we added the ^15^N solution. We added two midge and two mosquito larvae in each pitcher in the complete food web treatment at Fort Albany and nine midge and nine mosquito larvae in each pitcher in the complete food web treatment at Tom Swamp (*i.e.*, 1 midge+1 mosquito larva per ml of pitcher liquor). Unfed (control) pitchers for which we measured natural abundance of ^13^C and ^15^N also had complete food webs (pitcher liquor+midge+mosquito larvae).

Because of the dramatic size differences between plants at the two sites (**Table 1**), total N fed to each plant and food web manipulations (numbers of midge and mosquito larvae added to pitchers) differed at the two sites. Therefore, statistical analyses were conducted separately for each site.

### Harvest

Target pitchers were removed from the rest of the plant 3 or 72 hr after feeding with a stainless steel razor blade that was rinsed in 50% ethanol between cuttings. Pitcher liquor was transferred to a sealed sterile plastic tube and the pitcher was placed in a zip-lock plastic bag. Both were stored in a cooler with cold packs and taken immediately to the laboratory for processing. Pitchers were cut open longitudinally, washed thoroughly with tap water, rinsed with 0.5 mM CaCl_2_ to remove any amino acids from the surface [54], and finally rinsed three times with distilled-deionized water before being transferred to paper bags. Midge and mosquito larvae were removed from the pitchers with an eye dropper, transferred through three sequential baths of distilled-deionized water and stored in new sterile vials. Because of the small mass of larvae in each pitcher, larvae from the five replicates of each harvest × treatment combination were pooled into one composite larval sample. Plant and invertebrate samples were then oven-dried at 65°C for 48 h and then weighed.

### Isotopic analyses

Each pitcher and composite larval sample was ground to a fine powder in a stainless steel capsule with a stainless steel ball using a Wig-L-Bug mixer (Bratt Technologies, LLC., East Orange, New Jersey, USA). A 4-mg subsample of plant tissue or a 1-mg subsample of larvae was then placed into an 8×5 mm tin capsule (Elemental Microanalysis Mason, Ohio, USA) and combusted in a Costech ECS4010 Elemental Analyzer and DeltaPlus XP mass spectrometer at the University of New Hampshire to measure ^13^C/^12^C, %C, ^15^N/^14^N and %N concurrently. A reference standard (NIST 1515, NIST 1575a, or an internal tuna standard) was included after every five samples.

Recovery of added tracer in pitchers was calculated as:



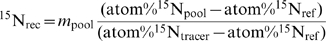
where ^15^N_rec_ = mass of ^15^N tracer recovered in the labeled N pool, *m*_pool_ = N mass of the total N pool, atom%^15^N _pool_ = atom percent ^15^N in the labeled N pool, atom%^15^N_ref_ = atom percent ^15^N in the reference N pool (non-labeled plants harvested at the end of the experiment), and atom%^15^N_tracer_ = atom percent ^15^N of the applied tracer [55].

### Statistical analysis

First, we measured ^15^N and ^13^C in pitchers to determine if *S. purpurea* could acquire intact amino acids. We emphasize that observing enrichment in ^13^C and ^15^N by itself does not provide evidence for acquisition of intact glycine or phenylalanine because both ^13^C and ^15^N may be acquired in products of microbial mineralization of amino acids. Rather, a comparison of the slope of excess ^13^C versus ^15^N (per gram dry mass of plant tissue) to the slope of the ^15^N source provides a conservative estimate of N acquired as amino acid [56]. Ratios below the expected slope ( = ^13^C∶^15^N ratio of the amino acids) indicate loss of ^13^C (e.g., respiration) and/or ^15^N acquisition after mineralization of labeled amino acid. Such an analysis should be undertaken within a few hours of the application of dual-labeled amino acids (hence our 3-hour harvest). We used ordinary least-squares linear regression analysis to compare the slope of ^13^C∶^15^N in pitchers fed **U-Gly** or **U-Phe** as their only N source with the expected slopes of ^13^C∶^15^N if glycine (expected slope = 2) or phenylalanine (expected slope = 9) were taken up intact within the first 3 hours after feeding.

**Figure 2:**
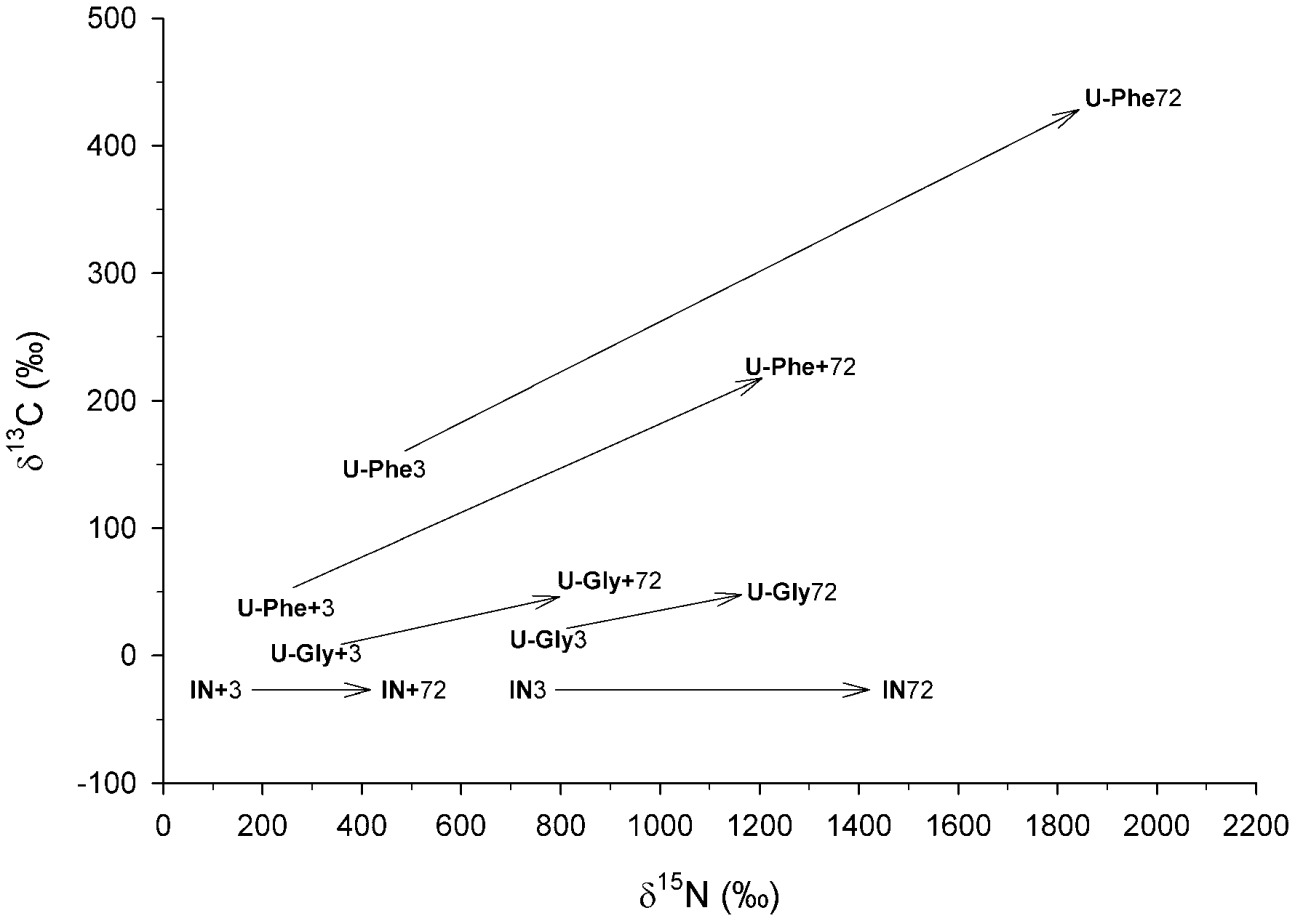
Enrichment of mosquito and midge larvae collected from *Sarracenia purpurea* pitchers. The arrows connect points showing enrichment after 3 and 72 hours of plants at Tom Swamp in Massachusetts that were fed ^15^N as ammonium nitrate (^15^NH_4_^15^NO_3_), uniformly-labeled (U-) ^13^C-^15^N-glycine or U-^13^C-^15^N-phenylalanine alone (IN, U-Gly, and U-Phe, respectively) or in combination (IN+, Gly+, and Phe+, respectively). Larvae in pitchers fed Phe or Phe+ had the highest ^13^C and ^15^N enrichment after 72 hours.

For all other analyses, we analyzed nitrogen uptake by pitchers as μg ^15^N per gram dry mass. Data were arcsin-square-root transformed to reduce heteroscedasticity. Although ^15^N enrichment was measured in midge and mosquito larvae (**Fig. 2**), analysis of variance with a main effect for the invertebrate food web manipulation revealed no significant differences in ^15^N uptake at each site for pitchers with and without the invertebrate food web (Fort Albany F_1,96_ = 0.137, *P* = 0.71; Tom Swamp F_1,94_ = 1.68, *P* = 0.20). Therefore, ^15^N uptake by pitchers was analyzed for each site separately using a fixed-effect two-way analysis of variance to test for differences in ^15^N uptake only as a function of the form of N fed to pitchers and harvest time. In these analyses, pitchers in the two food-web treatments were pooled within each N addition treatment. *A priori* contrasts of the N-form treatment at the 72-h harvest were used to compare plant N uptake across treatments and between sites at the end of the experiment.

## Results

### Uptake of intact amino acids

**Figure 3:**
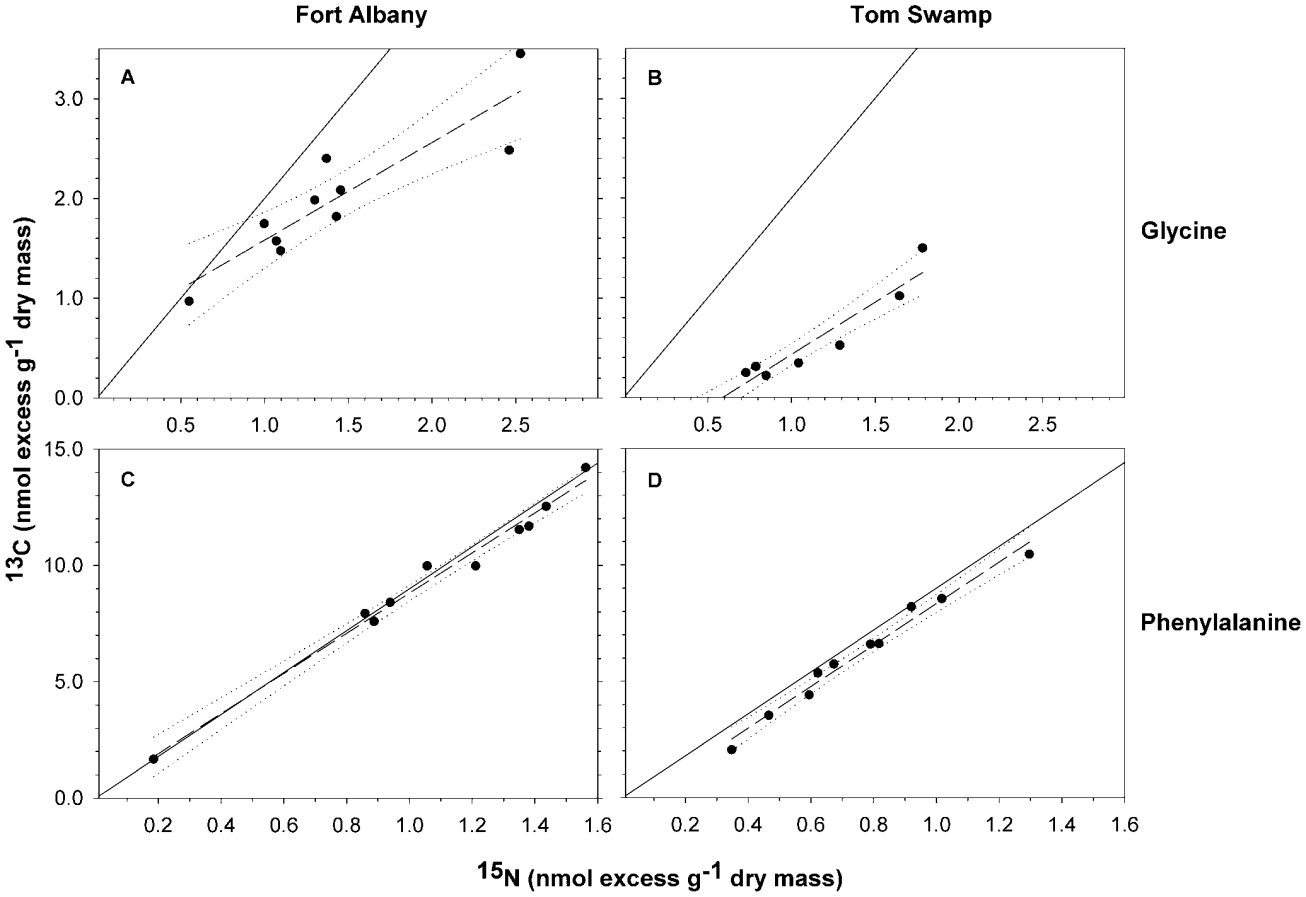
Concentration of ^13^C and ^15^N recovered in *Sarracenia purpurea* pitchers 3 h after feeding. Nitrogen solutions were applied into the pitcher leaves at Fort Albany in Ontario, Canada (left, panels A, C) and at Tom Swamp in Massachusetts, U S A (right, panels B, D) as uniformly-labeled (U-) ^13^C-^15^N-glycine (top row, panels A, B) or as U-^13^C-^15^N-phenylalanine (bottom row, panels C, D). Solid lines represent the ^13^C∶^15^N ratio of glycine (2∶1) or phenylalanine (9∶1); dashed-lines represent ordinary least squares regression (with 95% confidence intervals as dotted lines) for plant uptake.

Pitcher plants rapidly assimilated the two amino acids we fed to them. There was a significant positive relationship between tissue ^13^C and ^15^N for pitchers harvested 3-h after receiving only **U-Gly** at Fort Albany (*P* = 3.83×10^−4^; **Fig. 3A**) and Tom Swamp (*P* = 6.84×10^−6^; **Fig. 3B**). The slope of the ^13^C∶^15^N line at both sites was significantly less than 2 – the value expected if intact glycine was taken up directly – at both Fort Albany (slope = 0.98, 95% CI = 0.6–1.4) and at Tom Swamp (slope = 1.1, 95% CI = 0.8–1.3).

There was a highly significant positive relationship between tissue ^13^C and ^15^N for pitchers harvested 3-h after receiving **U-Phe** at both Fort Albany (*P* = 1.28×10^−8^; **Fig. 3C**) and Tom Swamp (*P* = 7.17×10^−8^; **Fig. 3D**). The slope of the ^13^C∶^15^N relationship for pitcher tissue at both sites did not differ from 9 – the value expected if intact phenylalanine was taken up directly – at both Fort Albany (slope = 8.6 with a 95% CI = 7.7–9.5; **Fig. 3C**) and at Tom Swamp (slope = 8.9 with a 95% CI = 7.8–10.0; **Fig. 3D**).

### Uptake of different forms of nitrogen

**Figure 4:**
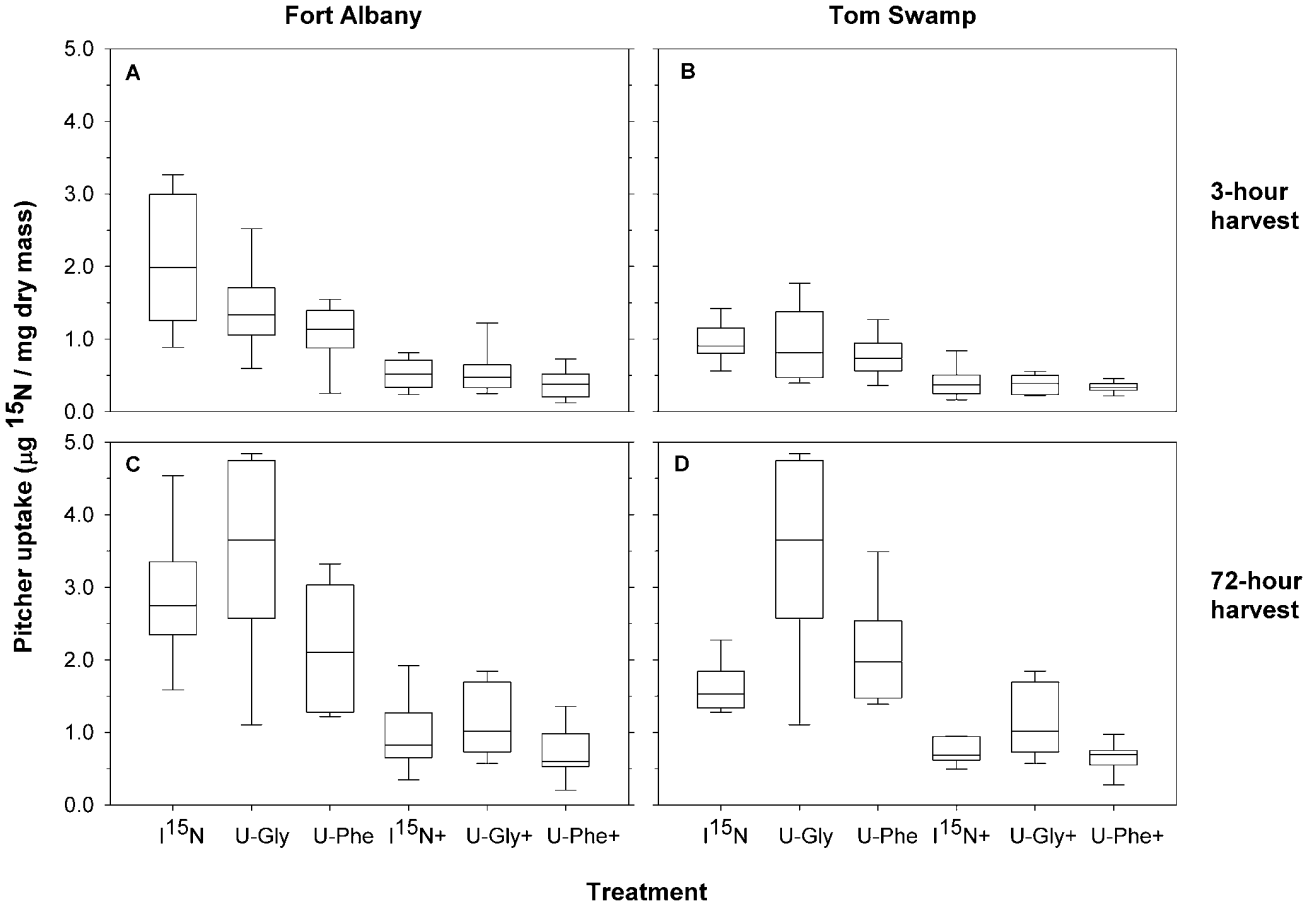
^15^N acquisition by *Sarracenia purpurea* pitchers supplied with multiple forms of nitrogen. Nitrogen was provided to pitcher leaves as ammonium nitrate (^15^NH_4_^15^NO_3_), uniformly-labeled (U-) ^13^C-^15^N-glycine or U-^13^C-^15^N-phenylalanine (IN, U-Gly, and U-Phe, respectively) or in combination (IN+, U-Gly+, U-Phe+, respectively) at Fort Albany, Ontario, Canada (left, panels A, C) and at Tom Swamp, Massachusetts, U S A (right, panels B, D). Uptake was measured after 3 hours (top, panels A, B) and 72 hours (bottom, panels C, D) in a pulse-chase experiment. The total amount of N provided to pitchers at Fort Albany was 0.022 mg, and was 0.101 mg N at Tom Swamp. Pitchers with and without the invertebrate food webs were pooled for these analyses because there were no significant differences in ^15^N uptake in the two food-web treatments (Fort Albany F_1,96_ = 0.137, *P* = 0.71; Tom Swamp F_1,94_ = 1.68, *P* = 0.20).

At each site, there were highly significant differences among treatments (Fort Albany: F_5,108_ = 41.8, *P*<0.0001; Tom Swamp: F_5,106_ = 47.0, *P*<0.0001) and harvest times (Fort Albany: F_1,108_ = 62.3, *P*<0.0001; Tom Swamp: F_1,106_ = 158.3, *P*<0.0001), and significant treatment × harvest time interactions (Fort Albany: F_5,108_ = 2.3, *P* = 0.05; Tom Swamp: F_5,106_ = 4.5, *P* = 0.0009; **Fig. 4**). At Fort Albany, pitchers acquired significantly more (*P* = 0.004) ^15^N from glycine than from phenylalanine when those forms were provided in isolation (**Fig. 4A**), and showed a similar trend when all three forms of N were available (*P* = 0.07; **Fig. 4C**). At Tom Swamp pitchers acquired similar amounts (*P* = 0.56) of N from glycine and phenylalanine when ON was provided in isolation (**Fig. 4B**) but tended to favour glycine when all forms of N were available (*P* = 0.08; **Fig. 4D**).

At Tom Swamp, pitchers took up significantly more N from either glycine or phenylalanine than from ammonium nitrate (*P* = 0.034 and *P* = 0.009, respectively) when only one form of ^15^N was provided (**Fig. 4D**). In contrast, at Fort Albany, pitchers took up similar amounts of ^15^N either the amino acids or the ammonium nitrate when only one form of ^15^N was available (*P* = 0.22 and *P* = 0.09, respective contrasts; **Fig. 4C**). At both sites, pitchers acquired similar amounts of ^15^N from the amino acids and ammonium nitrate when all three forms of N were provided simultaneously (*P*≥0.29). Finally, highly significant differences of ^15^N uptake were found for each form of N when it was provided in isolation relative to when it was provided in mixture (**Figs. 4C, 4D**).

## Discussion

Uptake of intact amino acids has been demonstrated for species in ecosystems ranging from alpine and arctic tundra to a subtropical rainforest and in ephemeral pools in the Namibian desert [15]. The use of a variety of forms of different nutrients could provide selective advantages to plants inhabiting nutrient-limited environments. Carnivorous plants, whose growth and reproduction are strongly limited by nutrient availability and that grow in extremely nutrient-poor habitats [19], [21] acquire organic nutrients as prey, but whether or not they can directly take up intact amino acids had not been studied previously.

Our data are consistent with the hypothesis that *S. purpurea* takes up amino acids directly. However, while this was clearly demonstrated for phenylalanine, *S. purpurea* either did not assimilate glycine directly or else it assimilated glycine but metabolized it more rapidly than our 3-hour sample could detect (**Fig. 3**). All pitchers fed **U-Gly** and **U-Phe** were highly enriched in ^13^C and ^15^N. Rapid enrichment of pitcher tissue with both ^13^C and ^15^N at a ^13^C∶^15^N ratio similar to that of the amino acid fed to the plant would suggest uptake of intact amino acids. Whereas pitchers fed **U-Phe** had ^13^C∶^15^N ratios similar to the expected value of 9, pitchers fed **U-Gly** were highly enriched in both ^13^C and ^15^N but their ^13^C∶^15^N ratio was significantly less than the expected value of 2. The results for acquisition of intact glycine, and comparison to phenylalanine, however, must be interpreted with caution [57], [58]. First, carbon respiration following acquisition of phenylalanine will have less effect on the ^13^C∶^15^N relationship than it would for glycine because of the greater amount of ^13^C acquired per unit phenylalanine (C∶N = 9) compared to glycine (C∶N = 2). Carbon acquired from glycine is rapidly catabolised [59], which can lead to slopes of ^13^C∶^15^N substantially below the expected value of 2 (**Figs. 3A, 3B**). This may have been exacerbated by the relatively small amounts of ^13^C- and ^15^N-enriched amino acids provided to pitchers; in order to avoid potential effects of excess N availability on the plant, added N represented <2% of the total N in the pitchers.

Furthermore, the substantial enrichment of pitchers (**Fig. 4**) and the differences in ^13^C and ^15^N enrichment of invertebrate larvae (**Fig. 2**) between pitchers fed **U-Gly** and **U-Phe** provides some additional support for intact glycine acquisition by *S. purpurea* pitchers under field conditions. As expected if plants preferentially acquire amino acids with low C∶N [60], the ^15^N enrichment of *S. purpurea* pitchers was generally greater for plants fed **U-Gly** than **U-Phe** (**Fig. 4**).

More detailed comparisons of enrichment of ^15^N in plant tissues of the different treatment groups suggests that *Sarracenia* should show preferential uptake of amino acids with lower C∶N ratios (such as glycine) than those with higher C∶N ratios (such as phenylalanine). After 72-h, ^15^N concentration in plants fed glycine alone was significantly greater than ^15^N concentration in plants fed phenylalanine alone at Fort Albany but not at Tom Swamp (**Fig. 4**). At both sites, uptake of ^15^N from glycine tended to be higher than uptake of ^15^N from phenylalanine when all three forms of N were available to pitchers (**Fig. 4**). Because Fort Albany has cooler air temperatures, a shorter growing season, and lower atmospheric N deposition than Tom Swamp, we interpret these results to suggest that pitchers at the Canadian site can maximize N uptake by taking up relative more amino acids with low C∶N ratios (*e.g.*, glycine) and avoid the energetically costly synthesis of new amino acids from IN plus a carbon skeleton [18]. In contrast, at Tom Swamp in Massachusetts, the climate is warmer, the growing season is longer, and IN is more readily available because of higher atmospheric deposition rates. Thus, direct uptake of low C∶N amino acids such as glycine may not be as important at Tom Swamp because suitable environmental conditions exist to synthesize amino acids from readily available IN plus available carbon.

These inferences are supported by our results showing greater ^15^N uptake from ON than from IN (energetic benefit) when each form was provided in isolation at Tom Swamp (**Fig. 4**). At the more N-limited Fort Albany site, however, ^15^N uptake by *S. purpurea* pitchers was similar for all three forms of ^15^N when each was provided in isolation. *Sarracenia purpurea* pitchers are open to the atmosphere, collecting rainwater as well as prey, and the difference in ON uptake between sites may represent a response to the higher atmospheric IN deposition at Tom Swamp. Similar preferential acquisition of the predominant forms of N in the local environment has been observed for plants in boreal forests [61], arctic tundra [62], [63], alpine meadows [35], and cold-temperate forests [64].

Finally, our results illustrate that the acquisition of any one form of N provided in isolation will exceed uptake of this form when multiple forms of N are made available to the plant simultaneously. At both sites and for each form of ^15^N supplied, uptake of ^15^N was significantly greater when only one form was made available compared to that same form of ^15^N provided in mixture. However, there were no significant differences in ^15^N uptake among the three forms when all forms of ^15^N were made available simultaneously (**Fig. 4**). This result highlights the versatility of N acquisition by *S. purpurea* because N uptake of any one particular form decreases when all three forms are available.

*Sarracenia purpurea* is one of only two species of North American pitcher plants (the other being *Darlingtonia californica*) in which a food web mineralizes captured prey and simultaneously competes with *S. purpurea* for N. Our results revealed no differences in ^15^N uptake between pitchers with and without higher trophic levels in their associated food webs. This result is consistent with previous results showing that the upper trophic levels in the *S. purpurea* microecosystem actively process detritus but that the activity of the microbial component of the food web ultimately determines N availability for *Sarracenia* [30].

Taken as a whole, our field experiments indicate versatility of N acquisition by this carnivorous plant and variability in N acquisition across a gradient of atmospheric deposition. These results are consistent with data reported from other N-limited environments [54], [65]. Similarly, there is pronounced spatiotemporal variation in the availability, form, quantity, and proportions of each form of N in bogs in general and in the *Sarracenia* microecosystem in particular. The growth of plants in eastern North American bogs is predominantly N-limited [33] but bogs have massive stores of ON in peat with high nutrient flux and organic production [6] and receive variable inputs of atmospheric IN deposition throughout the growing season. Additionally, pitcher plants collect varying amounts of organic and inorganic N via trapped prey. We suggest that the energetic benefits of direct and rapid (<3 h) acquisition of ON as intact amino acids allow *Sarracenia* to short-circuit the inorganic N cycle and to minimize potential bottlenecks in N availability because of the plant's reliance for N mineralization on a seasonally reconstructed food web [66] operating on irregular and infrequent seasonal pulses of prey capture [45], [67]. Experiments employing a greater range of N concentrations for a longer duration would improve our ability to determine the upper limit of N acquisition by *Sarracenia* and characterize the importance of the acquisition of intact amino acids to the N budget of this carnivorous plant.
